# Supplementary material for: A novel framework for assessing causal effect of microbiome on health: long-term antibiotic usage as an instrument
Source: Gut Microbes. 2025 Jan 23;17(1):2453616. doi: 10.1080/19490976.2025.2453616 (PMC11776458; doi:10.1080/19490976.2025.2453616)
Supplement: Supplemental Material [file KGMI_A_2453616_SM2369.zip › ABinstrument_final supplement fig.docx]

Supplementary figures


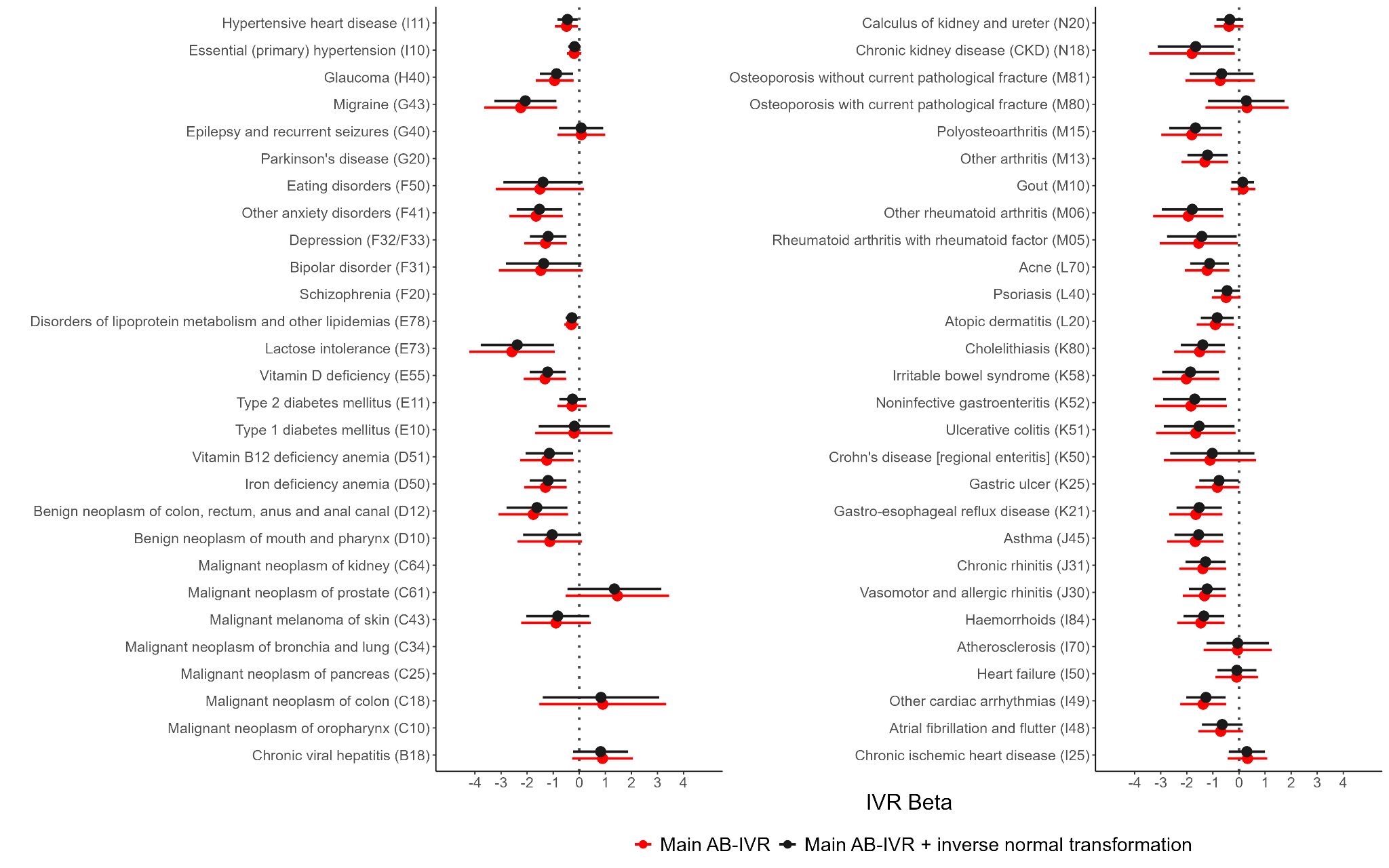


**Supplementary figure 1. Results of the main analysis for all the 56 studied diseases.** Colours represent different data transformations for the *Prevotella/Bacteroides* ratio. Sensitivity analysis results with the inverse normal transformation is shown in black.


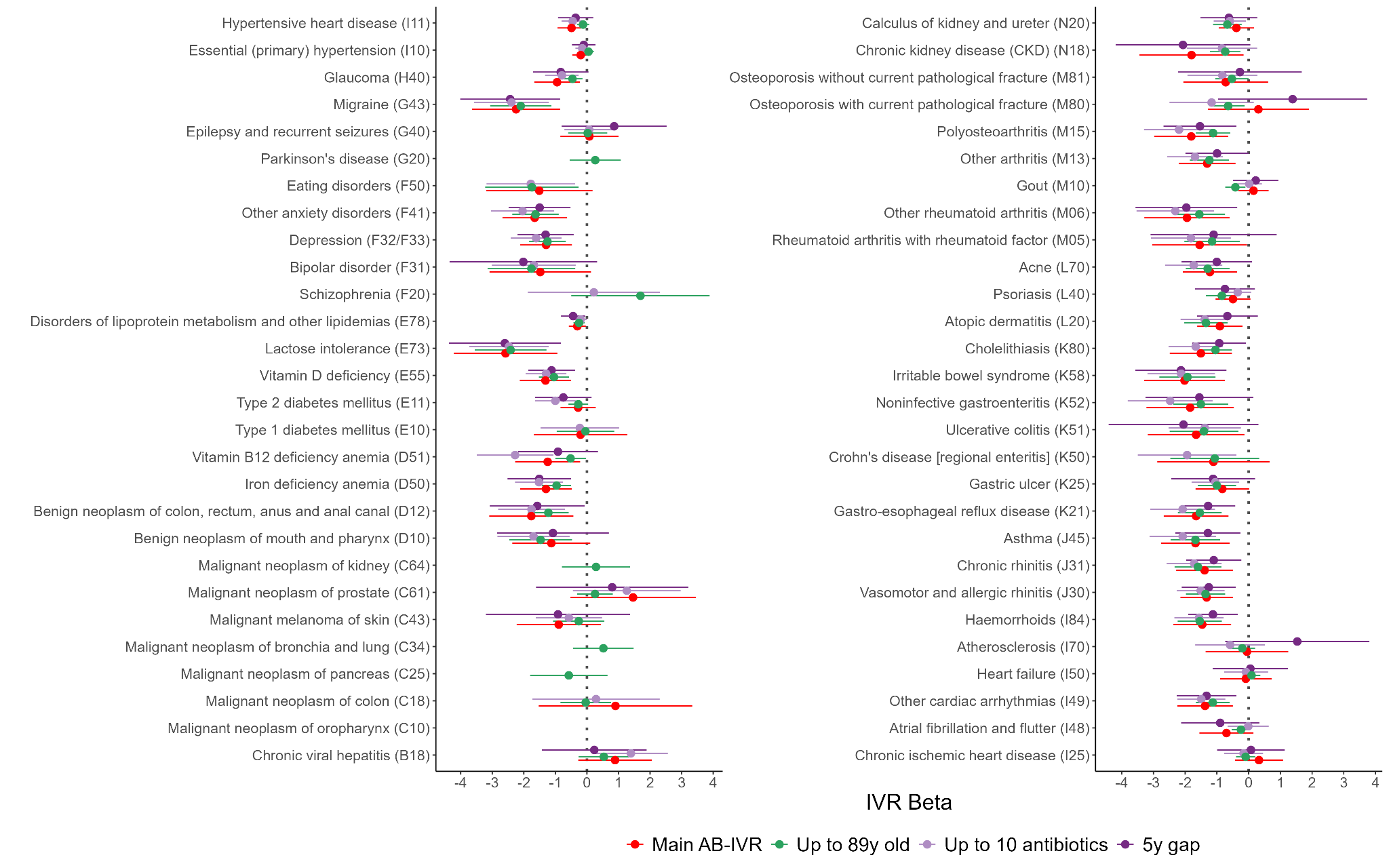


**Supplementary figure 2. Results of the sensitivity analysis analysis for all the 56 studied diseases.** Green shows the effect estimates, when age is filtered as 23-89; light-purple shows the effect estimates, when the maximum number of AB prescribed is 10; dark-purple describes the scenario where the first five years of incidence after the start of follow-up is considered as prevalent disease.


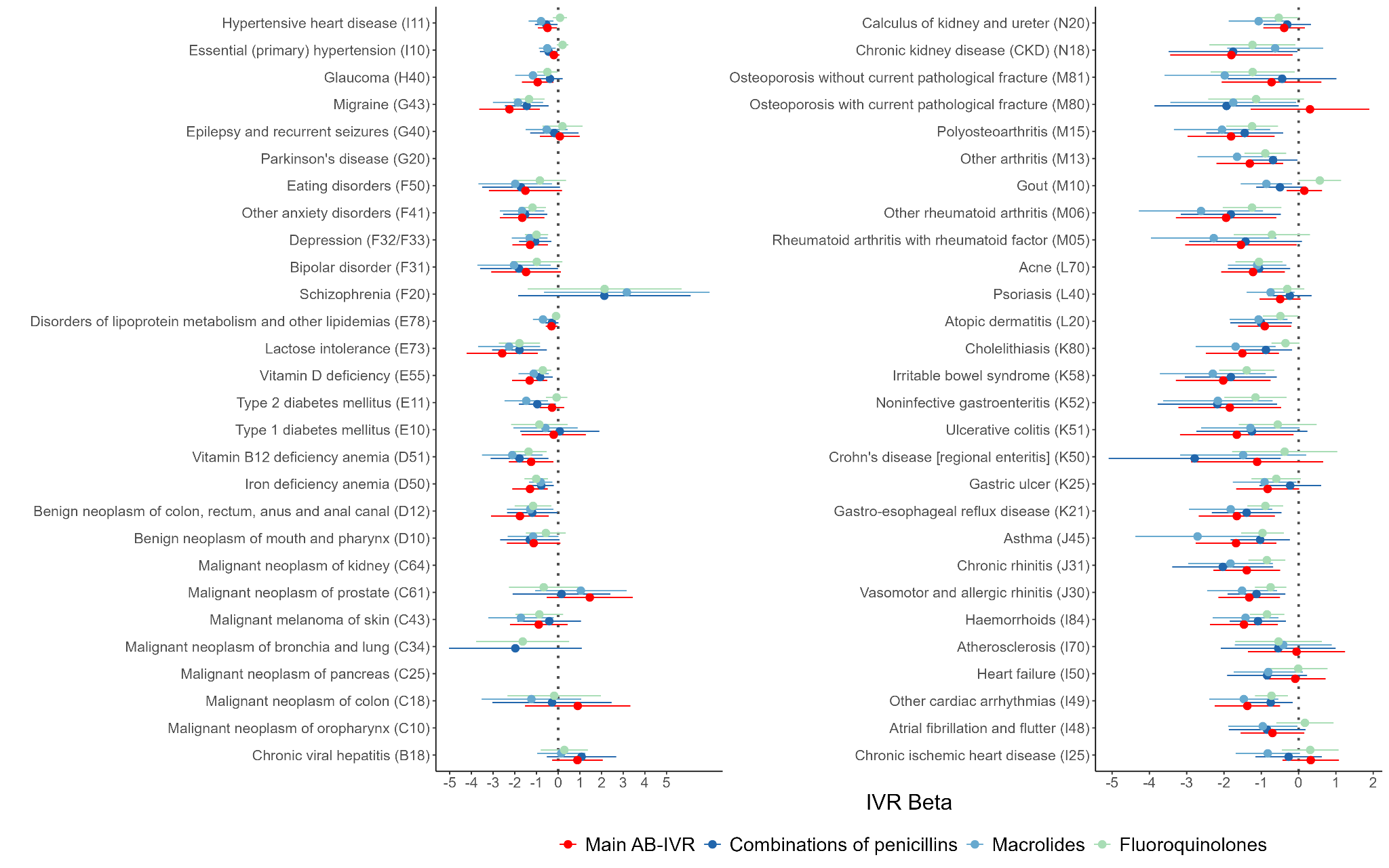


**Supplementary figure 3. Results of the sensitivity analysis for all the 56 studied diseases.** Information regarding subclasses of AB were used instead of the total amount of AB prescribed, whereas other settings were identical to the main analysis**.** Red corresponds to the main analysis with the antibiotics combined, dark blue corresponds to the class of penicillins (J01CR), light-blue corresponds to the class of macrolides (J01FA) and light-green corresponds to the class of fluoroquinolones (J01MA).


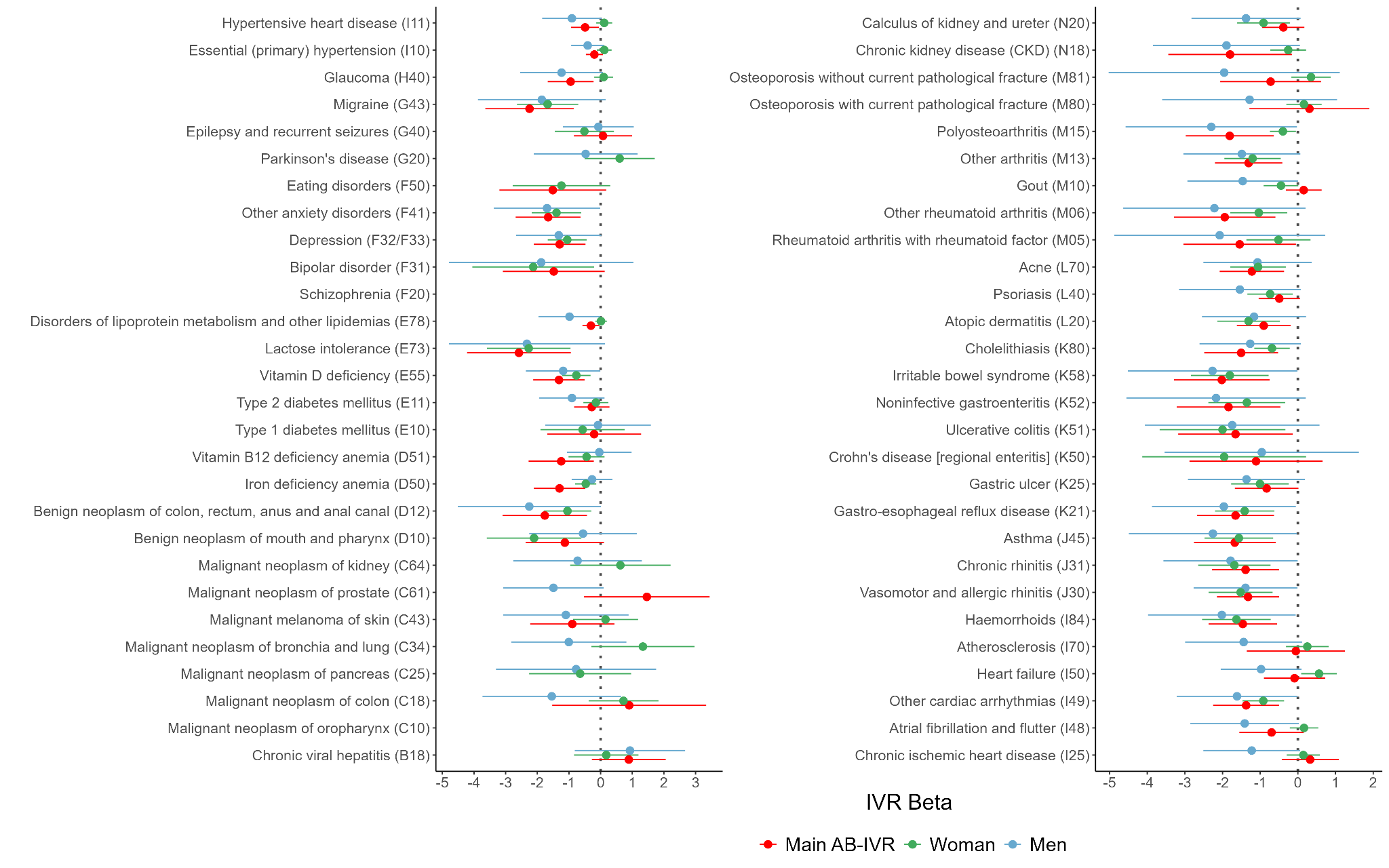
**Supplementary figure 4. Results of the sensitivity analysis for all the 56 studied diseases.** Sensitivity analysis in Women and Men subcohorts separately. Red corresponds to the main analysis, green corresponds to women aged 23-89, and blue corresponds to men aged 23-89.
